# Subregional 6-[^18^F]fluoro-ʟ-*m*-tyrosine Uptake in the Striatum in Parkinson's Disease

**DOI:** 10.1186/1471-2377-11-35

**Published:** 2011-03-23

**Authors:** Sayaka Asari, Ken-ichi Fujimoto, Akihiro Miyauchi, Toshihiko Sato, Imaharu Nakano, Shin-ichi Muramatsu

**Affiliations:** 1 Division of Neurology, Department of Medicine, Jichi Medical University, Tochigi, Japan; 2 WebNet Technology, Tochigi, Japan; 3 Utsunomiya Central Clinic, Tochigi, Japan

## Abstract

**Background:**

In idiopathic Parkinson's disease (PD) the clinical features are heterogeneous and include different predominant symptoms. The aim of the present study was to determine the relationship between subregional aromatic l-amino acid decarboxylase (AADC) activity in the striatum and the cardinal motor symptoms of PD using high-resolution positron emission tomography (PET) with an AADC tracer, 6-[^18^F]fluoro-ʟ-*m*-tyrosine (FMT).

**Methods:**

We assessed 101 patients with PD and 19 healthy volunteers. PD was diagnosed based on the UK Brain Bank criteria by two experts on movement disorders. Motor symptoms were measured with the Unified Parkinson's Disease Rating Scale (UPDRS). FMT uptake in the subregions of the striatum was analyzed using semi-automated software for region-of-interest demarcation on co-registered magnetic resonance images.

**Results:**

In all PD patients, FMT uptake was decreased in the posterior putamen regardless of predominant motor symptoms and disease duration. Smaller uptake values were found in the putamen contralateral to the side with more affected limbs. The severity of bradykinesia, rigidity, and axial symptoms was correlated with the decrease of FMT uptake in the putamen, particularly in the anterior part. No significant correlation was observed between tremors and FMT uptake.

**Conclusions:**

Decrease of FMT uptake in the posterior putamen appears to be most sensitive in mild PD and uptake in the anterior putamen may reflect the severity of main motor symptoms, except for tremor.

## Background

Cardinal motor symptoms such as bradykinesia, rigidity, and tremor in Parkinson's Disease (PD) become apparent after a depletion of dopamine in the striatum to approximately 20% of normal levels and a reduction in aromatic ʟ-amino acid decarboxylase (AADC) activity to 5%-20% of normal levels [1,2]. In PD, dopaminergic hypofunction in the striatum is not homogenous in association with the selective loss of ventral intermediate and lateral cell groups of the substantia nigra pars compacta that project to the posterior part of the striatum [3], although the reason for this selective vulnerability remains unknown.

Positron emission tomography (PET) is valuable for assessing altered dopamine function in PD. The first tracer used to visualize and assess the integrity of dopamine presynaptic systems was 6-[^18^F]fluoro-ʟ-dopa (FDOPA), a fluoro-analog of ʟ-dopa [4]. FDOPA is taken up into the dopaminergic axon terminals and decarboxylated by AADC before being trapped and stored in synaptic vesicles. FDOPA uptake is highly correlated with viable dopaminergic cells in neurotoxin-lesioned monkeys [5] and in postmortem human PD brains [6]. A shortcoming complicating the use of this agent, however, is that metabolites of FDOPA (such as 3-*O*-methyl-[^18^F]fluoro-ʟ-dopa, which is formed by the action of the ubiquitous enzyme catechol-*O*-methyl-transferase (COMT)) enter the brain and diminish image contrast. An alternative agent is the non-catecholic tracer 6-[^18^F]fluoro -ʟ-*m*-tyrosine (FMT). FMT is also a good substrate for AADC but is not metabolized by COMT; thus, FMT uptake has approximately twice the sensitivity of FDOPA uptake and more fully represents the extent of AADC activity [7-10].

To elucidate the relationship between the main motor symptoms of PD and subregional AADC activity in the striatum, we applied a semi-automated segmentation method for extracting putaminal subregions from high-resolution FMT PET images that were co-registered with 3.0-tesla magnetic resonance (MR) images.

## Methods

### Subjects and clinical evaluation

Our sample consisted of 101 patients with PD and 19 healthy individuals. PD was diagnosed clinically according to the UK PD Society Brain Bank criteria [11]. All of the patients had bradykinesia and at least one of the three features of PD: 4-6 Hz resting tremor, rigidity, and postural instability. All of the patients had asymmetric onset and showed a positive response to dopaminergic medication. None exhibited atypical symptoms such as severe gaze palsy or symptomatic dysautonomia. The control group included healthy individuals with no history of neurologic or psychiatric diseases.

Motor symptoms were evaluated using the motor examination part of the Unified Parkinson's Disease Rating Scale (UPDRS). Motor subscores were determined as follows: tremor (motor UPDRS: 20 + 21), bradykinesia (motor UPDRS: 23 + 24 + 25 + 26), rigidity (motor UPDRS: 22), and axial (motor UPDRS: 18 + 19 + 27 + 28 + 29 + 30 + 31). The mini-mental state examination (MMSE) was used to assess cognitive function.

This study was approved by the Institutional Ethics Committee of Jichi Medical University and all participants gave written informed consent.

### PET imaging

All patients stopped levodopa at least 16 h before PET. To increase the availability of the tracer, all subjects took 2.5 mg/kg of carbidopa (a peripheral AADC inhibitor) orally 1 h before FMT injection. Prior to the emission scan, a 10 min transmission scan was obtained for attenuation correction. Subsequently, 0.12 mCi/kg of FMT in saline was infused into an antecubital vein and a 30-90 min static three-dimensional acquisition was started simultaneously using a PET-CT (GEMINI GXL, Philips, Amsterdam, The Netherlands). Each subject also underwent 3.0-tesla MR imaging (Achieva 3.0 T, Philips) using an inversion recovery (IR) proton density (PD)-weighted pulse sequence to enhance the contrast of anatomical structures. The PET and MR imaging data were co-registered with a fusion processing program (Syntegra, Philips) to produce fusion images. This program provided manual and point-based image registration as well as automated methods of gray-value-based image registration, including a mutual information algorithm [12]. In addition, an adaptive level set of segmentation was used for coregistration of CT and MRI imaging data [13].

### Semi-automated region of interest analysis

Regions-of-interest (ROIs) in the putamen and caudate nucleus were defined in three dimensions (3-D) bilaterally on the co-registered MR images where the striatum was best visualized. The putamen and the head of caudate nucleus were delineated by manual inspection on the three to five adjacent MR planes that corresponded to those planes on the PET images. The putamen was then automatically divided into three parts in the rostrocaudal direction using dedicated software for ROI demarcation. The 3-D ROIs (volumes of interest, VOIs) were extracted automatically by connecting two-dimensional drawings on each plane using a linear interpolation algorithm for VOI outlines. For reference, cerebellar ROIs were also defined in 3-D and located bilaterally on the cerebellar cortex.

Striatal-to-cerebellum ratio (SCR) values of radioactivity counts were calculated in the 80-90-min frame for each structure, using bilaterally averaged cerebellar ROI data as the denominator. For subregional analysis of their association with major motor symptoms in the PD subjects, SCR values from the caudate nucleus and each part of the putamen were analyzed on the contralateral to the more affected side of limb.

### Statistical Analysis

For comparison of more than two groups, one-way analysis of variance (ANOVA) was used. When the one-way ANOVA was significant at *p *< 0.05, post-hoc comparisons were conducted using Scheffé's test. We examined the correlation of FMT uptake in each part of the putamen with disease duration, and with the symptoms of bradykinesia, tremor, rigidity, and postural instability assessed on UPDRS motor scores. Non-linear exponential regression analysis was applied to assess the relationship between FMT uptake and disease duration (Prism, GraphPad Software, La Jolla, CA). SCR values and the UPDRS scores were compared by Spearman's rank correlation coefficient test. *P *< 0.05 was considered to indicate a statistically significant difference.

## Results

### Characteristics of subjects

Demographic and clinical characteristics of the patients with PD and those of the control subjects are listed in Table 1 and Table 2. The mean ages of the PD patients (41 male and 60 female) and the control subjects (6 male and 13 female) were 64.0 years (SD 9.3) and 56.7 years (SD 11.1), respectively. A wide range of duration and severity of symptoms was represented among the patients. The mean duration of symptoms was 6.0 years (SD 4.4) and the mean UPDRS motor score was 30.3 (SD 16.0). The right side was more affected in 55 patients.

**Table 1:** Clinical Characteristics of the Subjects

Characteristics	PD	Normal Control	*p *value
Age, year, mean ± SD	64.0 ± 9.3	56.7 ± 11.1	0.005
Male/Female	41/60	6/13	0.542
MMSE	27 ± 2.6	29 ± 1.3	0.005

MMSE, Mini Mental State Examination.

Data are given as mean ± standard deviation (SD) values.

**Table 2:** Clinical Charasteristics of the PD patients

Symptom duration, year	6.0 ± 4.4
More affected side	Right 55/Left 46
Hoehn-Yahr stage, on	2.4 ± 0.9
Hoehn-Yahr stage, off	3.3 ± 1.1
UPDRS score	
Total motor	30.3 ± 16
Bradykinesia	9.86 ± 6.3
Rigidity	6.15 ± 3.8
Axial	9.54 ± 6.2
Tremor	4.80 ± 4.0

UPDRS, Unified Parkinson's Disease Rating Scale

Data are given as mean ± standard deviation (SD) values.

### Subregional analysis of FMT uptake

**Figure 1:**
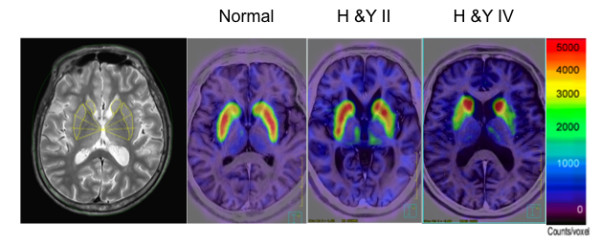
**Representative FMT-PET images of a healthy individual and PD patients**. FMT uptake declines asymmetrically in the early stages, mostly in the posterior putamen. Left: Regions-of-interest in the putamen. H &Y, Hohen and Yahr stage. The bar indicates the range of radioactive counts per voxel.

**Figure 2:**
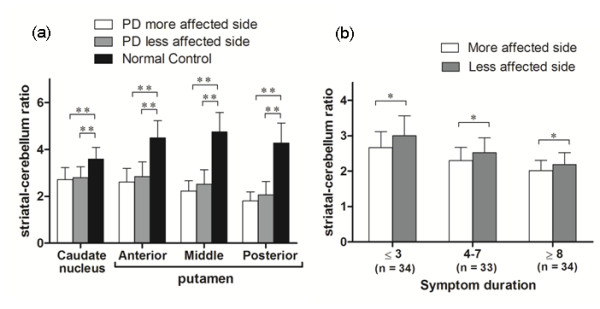
**FMT uptake in different subregions of the striatum**. Mean FMT uptake in different subregions of the striatum in normal control and PD patients (a). Comparison by side (b) shows persistant side-side asymmetry of putaminal uptake throughout the disease course. *P < 0.05, ** *P *< 0.01.

Figure 1 shows representative images of FMT uptake in a normal subject and in early- and late-stage PD patients. Among the patients, FMT uptake showed the most marked decrease in the posterior putamen, regardless of disease duration, but significant decrease was seen throughout the striatum compared with the healthy controls. There were significant differences between side (ipsi- vs. contralateral to the more affected limbs), region (anterior vs. posterior putamen), and diagnosis (healthy subjects vs. PD group) (*P *< 0.001) (Figure 2a). Asymmetry between the striatum of the more and less affected sides is preserved, but shows a decrease with disease progression (Figure 2b).

### Decline in FMT uptake with disease duration

**Figure 3:**
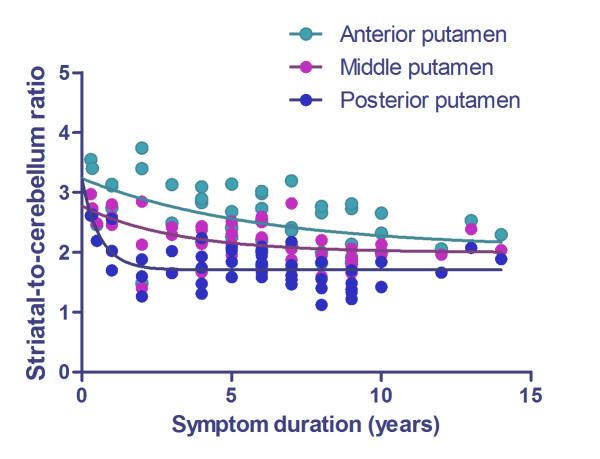
**Decline in FMT uptake with disease duration**. Scatter plots of FMT uptake against symptom duration in the putamen contralateral to the more affected limb in PD patients. Exponential decline is observed in all subregions of the putamen. Reduction of uptake is prominent at onset of the disease.

Figure 3 shows scatterplots of FMT uptake against symptom duration in three regions of the putamen contralateral to the more affected limbs. Because age-related factors such as age at onset of symptoms and age-related Alzheimer-type pathology may influence disease duration, we excluded elderly-onset patients (> 70 years old; *n *= 19) in this analysis. Exponential regression curves that best fitted the data for each of the three regions analyzed are superimposed on the figure. Between 10 and 15 years of symptom duration, the FMT for all three curves leveled off to constant values that showed a statistically significant difference between the anterior and posterior putamen (*p *< 0.001). In the control group, there was no significant difference in SCR of FMT uptake between younger (< 59 years old, *n *= 10) and older (≥ 60 years old, *n *= 9) subjects (putamen, *p *= 0.87; caudate, *p *= 0.81).

### Correlation of cardinal symptoms and FMT uptake

To minimize the possibility of including patients with alternative diagnoses, we analyzed patients who had cardinal motor symptoms for at least 3 years (*n *= 42). We obtained positive correlations between the severities of major motor symptoms: rigidity vs. axial symptoms (*r *= 0.68, *p *< 0.001), rigidity vs. bradykinesia (*r *= 0.56, *p *< 0.001), bradykinesia vs. postural instability (*r *= 0.54, *p *< 0.001), and tremor vs. bradykinesia (*r *= 0.39, *p *= 0.014). However, tremor did not have a significant relation with rigidity (*r *= 0.20, *p *= 0.20) or with axial symptoms (*r *= 0.12, *p *= 0.45). Axial symptoms, rigidity, and bradykinesia scores showed a correlation with FMT uptake in the contralateral putamen, with the highest correlation in the anterior putamen, but not in the contralateral caudate (Table 3). No significant correlation was evident between unilateral tremor scores from the most severely affected limbs and any of the striatal regions. To assess the potential influence of age, we analyzed older patients (> 60 years old; *n *= 25) separately and found similar correlations between major symptoms and FMT uptake (Table 4).

**Table 3:** Correlations of UPDRS scores and FMT uptake ratio values in the each part of the putamen

Putamen	Anterior	Middle	Posterior	Whole
Symptom duration, year	-0.52 (<0.001)	-0.56 (<0.001)	-0.51 (<0.001)	-0.58(<0.001)
Total motor score	-0.56 (<0.001)	-0.48 (0.002)	-0.41 (0.008)	-0.51 (0.001)
Bradykinesia	-0.54 (<0.001)	-0.53 (<0.001)	-0.44(0.005)	-0.55 (<0.001)
Rigidity	-0.50 (0.001)	-0.43 (0.006)	-0.37 (0.018)	-0.44 (0.005)
Axial	-0.60 (<0.001)	-0.51 (0.001)	-0.37 (0.016)	-0.50 (0.001)
Tremor	0.069 (0.658)	0.085 (0.587)	0.015 (0.925)	0.050 (0.747)

Data are given as *r *(*p*) values. These values were calculated by Spearman's rank correlation coefficient test. UPDRS motor score in off-medication state was evaluated in 42 subjects.

**Table 4:** Correlations of UPDRS scores and FMT uptake ratio values in the each part of the putamen in elder patients

Putamen	Anterior	Middle	Posterior	Whole
Symptom duration, year	-0.70 (<0.001)	-0.63 (<0.005)	-0.45 (<0.05)	-0.70(<0.001)
Total motor score	-0.56 (<0.01)	-0.50 (<0.05)	-0.37 (0.07)	-0.49 (<0.05)
Bradykinesia	-0.46 (<0.05)	-0.46 (<0.05)	-0.34(0.08)	-0.46 (<0.05)
Rigidity	-0.46 (<0.05)	-0.39 (0.05)	-0.31 (0.12)	-0.37 (0.06)
Axial	-0.69 (<0.001)	-0.59 (<0.01)	-0.45 (<0.05)	-0.58 (<0.01)
Tremor	0.26 (0.21)	0.12 (0.58)	0.06 (0.77)	0.14 (0.51)

Data are given as *r *(*p*) values. These values were calculated by Spearman's rank correlation coefficient test. UPDRS motor score in off-medication state was evaluated in 25 subjects.

## Discussion

Idiopathic PD is defined as a synucleinopathy in which Lewy bodies, pathological aggregations of the synaptic protein α-synuclein, are found in the dopaminergic neurons in the substantia nigra [14,15]. A reduction of dopamine in the striatum is a consistent finding in PD, although the clinical features are heterogeneous and include different predominant symptoms (resting tremor, bradykinesia, rigidity, or postural instability and gait disorder) with different rates of progression, and with or without dementia [16-19]. PET imaging is a valuable tool for assessing altered dopaminergic function in the striatum in PD. While FDOPA is suitable for assessing the metabolism of levodopa, FMT is superior for estimating AADC activity because it enables the production of higher-quality brain images [7,20-22]. The high resolution of FMT-PET images enables analysis of dopaminergic presynaptic changes in each subregion of the striatum.

In the present study, FMT uptake in PD was reduced in the putamen, particularly in the posterior part. The anterior-to-posterior gradient of the uptake decrease in the putamen persisted to the advanced stage of PD. These results are consistent with those of previous reports that used other tracers of presynaptic dopaminergic terminals, and are considered to reflect the selective degeneration of nigrostriatal pathways that project into the posterior part of the putamen [23-25]. The lowest value of FMT uptake was observed in the posterior part of the putamen contralateral to the more affected limbs, even in the early stage of the disease. Because we analyzed regions in the posterior one-third of the putamen on high-resolution images, it is unlikely that the decreases in uptake were caused by partial volume effects, which may arise from placement of a small ROI on inaccurately co-registered images.

Post-mortem investigations of PD demonstrate that the rate of decrease of nigral neurons is rapid in the initial stage of the disease: approximately 40%-50% are lost in the first decade, possibly with a slower rate of degeneration later on, to finally approach a normal age-related linear decline [26]. In the present study, loss of FMT was well fitted to symptom duration using a single exponential approximation. The exponential model provided a better fit than a linear model, indicating that the rate of decline in FMT uptake in the contralateral putamen was faster at the beginning of the disease and slowed down as the disease progressed, in agreement with the results of previous studies that used radiotracers for imaging nigrostriatal nerve terminals [23-25]. Because we performed cross-sectional analysis in the present study, and because all of the participants were on medication, the data do not provide accurate information regarding the natural course of the disease, even if PET measurements were taken in off-medication state. Even so, the present data are important for assessing the progression of dopaminergic hypofunction in the striatum under optimal medical treatment, and can provide the basis for the development of even better therapeutic strategies [27,28].

We applied striatal count ratios to analyze the relationships between subregional putaminal FMT uptake and clinical symptoms. Striatal count ratios using the cerebellum as the denominator have a strong correlation with striatal uptake constants (Ki values) [29,30]. The present FMT-PET study showed a significant correlation between cardinal motor symptoms (rigidity, bradykinesia, and axial symptoms) and uptake of the tracer in the putamen, and no significant correlation was found between tremor score and FMT uptake. These findings are consistent with the results of previous PET studies [31-33]. The clinical correlations were more significant in the anterior part of the putamen than in the posterior part, possibly reflecting a floor effect for the uptake of FMT in the posterior part of the putamen, where the decrease was severe even in the early stage of the disease.

The pathophysiological mechanism of tremor is not fully understood [34]. Tremor does not respond to L-dopa as well as do bradykinesia and rigidity. The fact that stereotactic lesion or deep brain stimulation of the ventral intermediate nucleus (Vim) of the thalamus successfully improves tremor indicates a strong association between non-dopaminergic thalamic and cerebellar systems, and tremor generation [35,36].

## Conclusions

Our results indicate that FMT-PET is useful for evaluating PD patients from the early stage of the disease and for studying the relationship between AADC activity and various clinical features. Decrease of FMT uptake in the posterior putamen appears to be most sensitive in mild PD, and uptake in the anterior putamen may reflect the severity of main motor symptoms, except for tremor. These data provide an important baseline for evaluating the effects of surgical interventions, such as gene therapy for PD.

## Competing interests

The authors declare that they have no competing interests.

## Authors' contributions

SA participated in designing the study, data collection, conducted the statistical analyses, interpreted data and drafted the first manuscript.

KF participated in data collection and interpretation of data.

AM participated in data collection and interpretation of data.

TS participated in data collection and interpretation of data.

IN participated in designing the study and interpretation of data.

SM conceived the study, participated in its design, data collection, interpretation of data and drafting the manuscript.

All authors read and approved the final manuscript.

## Pre-publication history

The pre-publication history for this paper can be accessed here:

http://www.biomedcentral.com/1471-2377/11/35/prepub
